# Evolution of the Northern Rockweed, *Fucus distichus*, in a Regime of Glacial Cycling: Implications for Benthic Algal Phylogenetics

**DOI:** 10.1371/journal.pone.0143795

**Published:** 2015-12-02

**Authors:** Haywood Dail Laughinghouse, Kirsten M. Müller, Walter H. Adey, Yannick Lara, Robert Young, Gabriel Johnson

**Affiliations:** 1 Department of Botany, MRC-166, National Museum of Natural History, Smithsonian Institution, Washington, DC 20013–7012, United States of America; 2 Department of Biology, University of Waterloo, Waterloo, ON, N2T 2T4, Canada; 3 Centre for Protein Engineering, University of Liège, Sart-Tilman, B-4000 Liège, Belgium; 4 Department of Botany and Laboratories of Analytical Biology, Smithsonian Institution Museum Support Center, Suitland, MD 20746 United States of America; University of Pennsylvania, UNITED STATES

## Abstract

Northern hemisphere rockweeds (*Fucus*) are thought to have evolved in the North Pacific and then spread to the North Atlantic following the opening of the Bering Strait. They have dispersed and widely speciated in the North Atlantic and its tributary seas. *Fucus distichus* is likely near the ancestral member of this genus, and studies have shown that there are several species/subspecies in this complex (i.e. *F*. *evanescens* and *F*. *gardneri*). We used phylogenetic and haplotype analyses to test the phylogenetic relationships and biogeography of *F*. *distichus*. Our data and subsequent analyses demonstrate that, unlike previous studies that lacked samples from an extensive geographical area of the Arctic and Subarctic, there is a distinct Arctic haplotype that is the source of subspecies in both the North Pacific and North Atlantic. *Fucus distichus* occupies a low tide zone habitat, and in Arctic/Subarctic regions it is adapted to the severe stress of sea ice coverage and disturbance during many months per year. We hypothesize that the very large geographic area of Arctic and Subarctic rocky shores available to this species during interglacials, supported by large Arctic/Subarctic fringe areas as well as unglaciated refugia during glacial cycles, provided a robust population and gene pool (described by the Thermogeographic Model). This gene pool dilutes that of the more fragmented and area-limited Temperate/Boreal area populations when they are brought together during glacial cycles. We suggest that similar subspecies complexes for a variety of Arctic/Subarctic shore biota should be examined further in this context, rather than arbitrarily being split up into numerous species.

## Introduction

It is widely supported by palaeontological evidence [1–4] that the dominant Arctic fauna evolved in the North Pacific and entered the Arctic Ocean with the opening of the Bering Strait from 3.5–5 MYA. Although significant direct palaeophycological evidence is lacking, the single predictive biogeographical model [5], based on marine algae, suggests that Arctic seaweeds would have had the same origin as these fauna. Nevertheless, it has been argued that the Arctic flora present a paradox [1], deriving instead from the Atlantic Ocean. More recently, a number of Arctic endemic red seaweeds have been shown to have North Pacific origins [6], and the ancestors of the northern rockweed *Fucus* are widely accepted to have originated in the North Pacific [1,2] and spread to the North Atlantic after the opening of the Bering Strait. Based on mitochondrial DNA (mtDNA) including the intergenic spacer (IGS) and cytochrome oxidase 1 (CO1) gene applied to Holarctic *Fucus* populations, Coyer et al. [7] concluded that at least two separate colonization events of *F*. *distichus* L., from the Pacific to the Atlantic, likely occurred since the Last Glacial Maximum (LGM). The transit and subsequent diversification of *Fucus* from Pacific to Atlantic are late Pliocene and Pleistocene events. In this paper we discuss *F*. *distichus*, the single species of the group to become well-established in the Arctic and warmer fringe regions, with genetically modified populations due to repeated glacial periods.

The *Fucus distichus* ‘complex’ has a long history of systematic confusion with its relation to *F*. *evanescens* C. Agardh and *F*. *gardneri* P. C. Silva, based on ecology, morphology, and biogeography. *Fucus distichus* was named by Linnaeus in 1767 [8], but without a type locality. C. Agardh (1821) based *F*. *evanescens* on North Pacific syntype localities (Sakhalin and Kamchtka, Russia) and Powell (1957) recognized a subspecies *F*. *distichus* subsp. *evanescens* [8]. *Fucus gardneri* is a nomen novum for the illegitimate *F*. *furcatus* C. Agardh with its type locality in Unalaska (AK, USA). Algaebase [8] treats *F*. *distichus* and *F*. *evanescens* as distinct taxa with *F*. *gardneri* being a synonym of *F*. *distichus*. Gabrielson et al. [9] initially recognized *F*. *distichus* subsp. *evanescens* (C. Agardh) Powell, with *F*. *gardneri* a synomym, however Gabrielson et al. [10] only recognizes *F*. *distichus* from Oregon northward to SE Alaska, based on the results of Coyer et al. [11]. where they conducted a molecular-based phylogeny of the genus *Fucus* using the ITS, mtDNA 23S, and mtDNA spacer. These loci could not decipher among *F*. *distichus* (including the different subspecies and formae), *F*. *evanescens*, and *F*. *gardneri*, thus determining all as a unique ‘Lineage 1A’ in which these ‘species’ formed a polytomy. These data indicated that these were not true species, but in fact, different forms of the same species with the authors suggesting uniting all taxa into *F*. *distichus*. Based on these results, The only species of *Fucus* in Wilce & Dunton [12] is *F*. *distichus*, present in the northeast Atlantic and the North Pacific Ocean and southeast Alaska.

**Fig 1:**
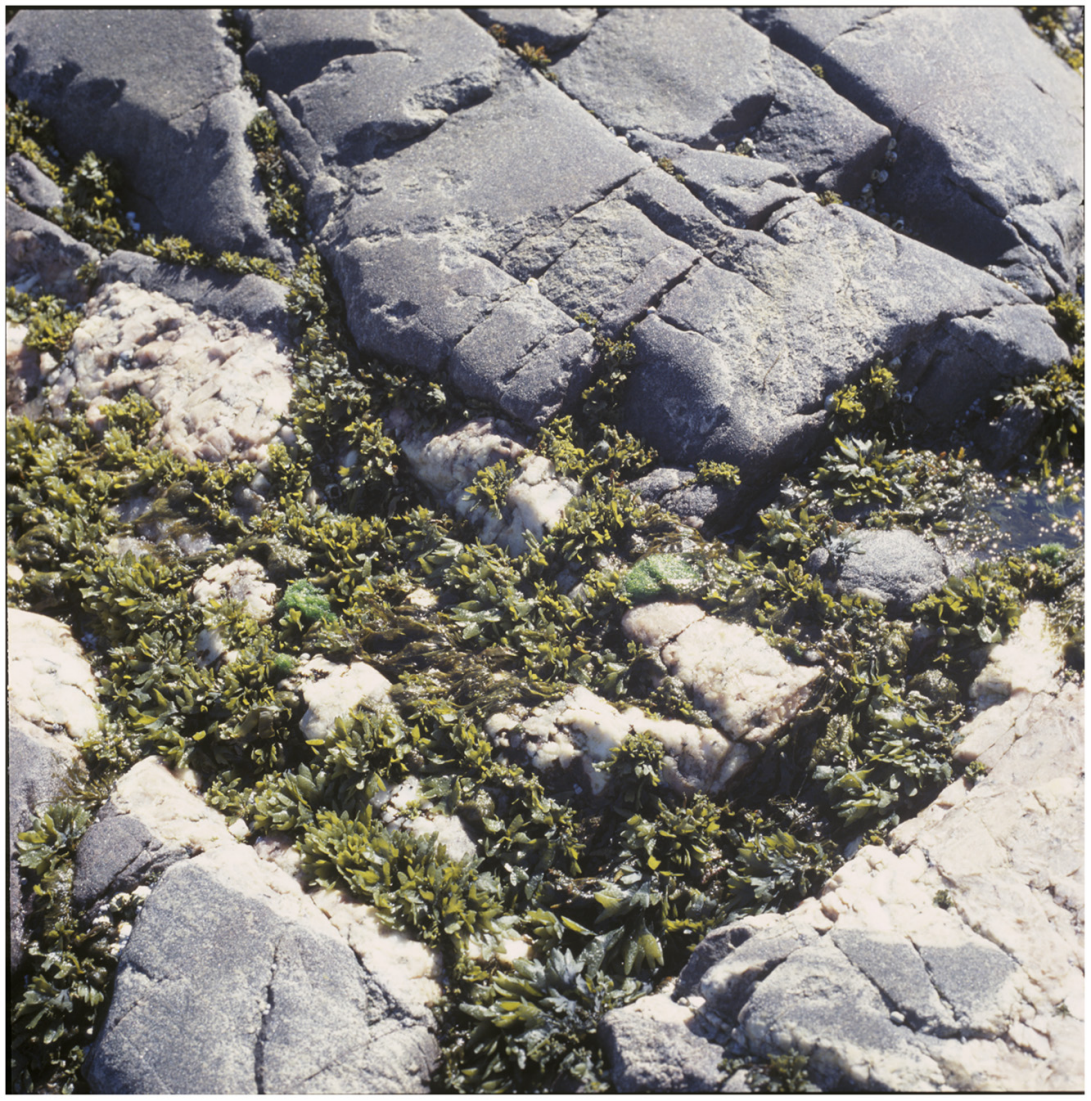
A dense patch of a small (dwarf) form of *Fucus distichus* in a lower intertidal crevice on a shore fully exposed to the Labrador Sea. This shore is scoured by dense, and sometimes moving sea ice from December through April of most years. Between storms and open water intervals, this shore is encased in an extensive ice foot. Battle Harbour, Labrador. Photo: W. Adey.

*Fucus distichus* extends southwards into boreal and mixed boreal/Subarctic regions [5], though it is a Subarctic species over most of its range. It successfully maintains populations in regions characterized by extensive sea ice throughout much of the year, despite having a low tidewater line habitat on rocky shores (lowermost intertidal, infralittoral, and uppermost sublittoral) [13]. In the Subarctic and low Arctic, *F*. *distichus* survives abundantly under shore-fast ice during long winter intervals. It is typically the dominant alga in the infralittoral of protected and intermediate exposure shores throughout the Subarctic [14]. Even on exposed shores, where ice scouring is extensive, it survives in smaller numbers by overwintering in crevices using a dwarf form that largely escapes ice scour (Fig 1).

There were at least eleven major northern hemisphere glaciations (during the Pleistocene, from approximately 2.6 MYA to 11,700 years BP) sufficient to have isolated Atlantic populations, with inter-glacial intervals that would have allowed new passage and population re-connection. As Adey & Hayek [14] explore, during glacial-interglacial transitions, high sea levels (prior to glacial rebound of the depressed earth’s crust) and periods of higher than current temperatures, may have allowed extensive colonization of *Fucus distichus* populations across the rocky Canadian Arctic Archipelago at times during each interglacial. In addition, large areas of rocky coast, both within the Canadian Arctic Archipelago, and on the fringes of the continental ice sheets, remained unglaciated [15].

The first North Atlantic colonization of *Fucus distichus* through the Canadian Arctic Archipelago from the North Pacific was likely on the abundant Subarctic rocky shores of the northwestern North Atlantic (Canadian Maritimes and Gulf of Maine). Colonization across northern Asia was less likely, since several very large rivers with abundant soft sediments characterize much of the 10,000 km-long Asian Arctic Coast. There is only a single large river in North America, the Mackenzie River, flowing northwards to Arctic shores. The resulting soft sediment shore of the Mackenzie River delta and adjacent North Slope of the Alaskan shores may well have provided more of a barrier to the dispersal of *F*. *distichus* than the interglacial climate of the Canadian Arctic Archipelago. As *Fucus distichus* thrives on rocky shores that are routinely occupied with sea ice for many months each winter [13], it is probable that this species occurs in a broken band through the entire low Arctic of the Canadian Archipelago during the current interglacial, as well as previous interglacials. Coyer et al. [7] had a considerable collection of North Pacific and especially northeastern North Atlantic *Fucus distichus* samples for their analysis. However, they lacked samples from the Canadian low Arctic and high Arctic, and had few Subarctic samples.

Historically, habitat characteristics and morphological characteristics were utilized to describe species within the genus *Fucus*. Nevertheless, *Fucus distichus* is known for its phenotypic plasticity, varying morphologically according to ecological factors/stimuli [16–17], such as desiccation, currents, frost, light, wave action, temperature, grazing, and due to geographical area and life history [18–19]. This has often led to erroneous identifications of field material. Furthermore, molecular data have shown that there is no resolution among *Fucus distichus*, *F*. *evanescens*, and *F*. *gardneri*, indicating that these are only one species [11].

In this paper, we report on the mtDNA intergenic spacer (mtDNA-IGS), the internal transcribed spacer region of the nuclear rDNA (nrDNA-ITS-1), and aspects of ecology and phylogeography, applied to a large collection of *Fucus distichus*, ranging from the northeastern North Pacific to the northwestern North Atlantic, but particularly concentrated in the Subarctic northwestern North Atlantic and through the Canadian Arctic Archipelago. We show that in addition to a North American West Coast population (classically referred to as *Fucus gardneri*); there are at least three genetically separate populations. With these data, we focus on: 1) discerning the ancestral haplotype in *Fucus distichus* and 2) the question of ‘species’ vs. ‘populations’ within the *Fucus distichus* complex. Understanding the control exerted on the low intertidal and uppermost subtidal seaweed habitat by seasonal sea ice, as well as the repeated longer-term separation of populations by Pleistocene Continental glaciation, is critical to the on-going species vs. populations/subspecies debate. A large number of new seaweed species are currently being created with little understanding of biological vs. biogeographic factors. We propose that all of these populations (‘*Fucus evanescens*’ C. Agardh, ‘*F*. *distichus*’, and ‘*F*. *gardneri*‘) may be able to interbreed and form a complex of regional subspecies developed due to periodic Arctic continental glaciation that closed off and fragmented Arctic gene flow for the species for 10–40 ky at each glaciation.

## Materials and Methods

### Field collection and sample sites

Samples of the *Fucus distichus* ‘complex’, *F*. *vesiculosus* L., and *F*. *serratus* L. were collected from the Pacific, Arctic and Atlantic Oceans and corresponding location names and coordinates of these samples are listed in Table 1. They were identified in the field using morphological and reproductive characters according to Sears [20]. Altogether, fifty-two specimens were collected from high to mid-intertidal areas predominantly along shorelines of rocky substrates from cold-water marine environments and also from the subtidal.

**Table 1:** Sampling location, information, and GenBank number for *Fucus* collected during the study.

Name in Tree	Taxon	Location	Latitude	Longitude	Haplotype	Collector/ Source	Accession #
		**Arctic and Subarctic**					
CA-Man1	*F*. *distichus*	Churchill, Manitoba, CAN	58°47' N	94°7' W	H18	this study	KT306695
CA-Lab1	*F*. *distichus*	Adlavik Harbour, Labrador, CAN	55°1' N	58°50' W	H18	this study	KT306700
CA-Lab2	*F*. *distichus*	Punchbowl, Labrador, CAN	53°15' N	55°45' W	H18	this study	KT306699
CA-Man2	*F*. *distichus*	Churchill, Manitoba, CAN	58°47' N	94°7' W	H18	this study	KT306698
CA-Lab3	*F*. *distichus*	Windy Tickle Harbour, Labrador, CAN	55°46' N	60°22' W	H17	this study	KT306701
CA-Nfld1	*F*. *distichus*	Musgrave ledges, NF, CAN	49°27' N	53°58' W	H19	this study	KT306713
CA-Lab4	*F*. *distichus*	E. Kingitok Island, Labrador, CAN	55°26' N	59°53' W	H19	this study	KT306714
AY659893	*F*. *evanescens*	Nuuk, Greenland	64°11' N	51°24' W	H19	Coyer	
CA-Lab5	*F*. *distichus*	Venison Tickle, Labrador, CAN	52°58' N	55° 47' W	H1	this study	KT306719
CA-Lab6	*Fucus sp*.	Baker Island, Labrador, CAN	56°32' N	61°9' W	H1	this study	KT306718
US-ME1	*F*. *distichus*	Shag Rocks, Grand Manan Channel, Maine, USA	44°44' N	67°6' W	H6	this study	KT306715
US-ME2	*F*. *distichus*	Shag Rocks, Grand Manan Channel, Maine, USA	44°44' N	67°6' W	H6	this study	KT306716
CA-Nfld2	*F*. *distichus*	Fishing Point, St. Anthony, NF, CAN	51°21' N	53°13' W	H1	this study	KT306717
AY659895	*F*. *evanescens*	DeCourcy Is., British Columbia, CAN	49°8' N	123°48' W	H1	Coyer	
AY659885	*F*. *evanescens*	Appledore Island, Maine, USA	42°59' N	70°37' W	H7	Coyer	
AY659894	*F*. *evanescens*	Vatnsleysa, Iceland	64°3' N	22°6' W	H21	Coyer	
US-ME4	*F*. *distichus*	Shag Rocks, Grand Manan Channel, Maine, USA	44°44' N	67°6' W	H1	this study	KT306720
US-ME5	*F*. *distichus*	Shag Rocks, Grand Manan Channel, Maine, USA	44°44' N	67°6' W	H1	this study	KT306721
CA-NWT1	*F*. *distichus*	Cornwallis Is., North West Territories, CAN	74°39' N	94°22' W	H16	this study	KT306692
AY659896	*F*. *gardineri*	De Courcy Is., British Columbia, CAN	49°8' N	123° 48' W	H8	Coyer	
CA-Nvt1	*Fucus* sp.	Assistance Bay, Cornwallis Island, Nunavut, CAN	74°39' N	94°21'36" W	H1	this study	KT306691
CA-Nvt2	*Fucus* sp.	Beechy Island, Nunavut, CAN	74°42' N	91°48'36" W	H1	this study	KT306688
CA-Nvt3	*Fucus* sp.	Resolute, Nunavut, CAN	74°40'48'' N	94°54' W	H1	this study	KT306689
CA-Nvt4	*Fucus* sp.	Hudson Bay, Nunavut, CAN	NA	NA	H1	this study	KT306690
AY659884	*F*. *distichus*	NA	NA	NA	H20	Coyer	
		**North Pacific (+Iceland)**					
CA-BC4	*F*. *distichus*	Botany Bay, British Columbia, CAN	48°32 'N	124°27' W		this study	KT306724
US-WA1	*Fucus* sp.	Teahwit, Washington, USA	47°55' N	124°38' W	H14	this study	KT306685
AY659897	*F*. *gardneri*	Juneau, Alaska, USA	58°20' N	124°38' W	H14	Coyer	
AY941092	*F*. *gardneri*	Garðskågi, Iceland	64°4' N	22°42' W	H15	Coyer	
AY659889	*F*. *evanescens*	Muroran, Hokkaido, Japan	42°21' N	140°59' E	H13	Coyer	
AY659890	*F*. *evanescens*	Muroran, Hokkaido, Japan	42°21 'N	140°59' E	H10	Coyer	
CA-BC5	*Fucus* sp.	Parksville, British Columbia, CA	49°19' N	124°16' W		this study	KT306722
AY941094	*F*. *gardneri*	Prince William Sound, Alaska, USA	60°29' N	147°44' W		Coyer	
CA-BC6	*Fucus* sp.	Whiffen Spit Park, British Columbia, CAN	48°21' N	123°44' W		this study	KT306726
US-WA2	*Fucus* sp.	Kalaloch Beach, Washington, USA	47°38' N	124°23' W	H12	this study	KT306696
US-WA3	*Fucus* sp.	Ruby Beach, Washington, USA	47°38' N	124°23' W	H11	this study	KT306693
AY659887	*F*. *distichus*	Auke Bay, Alaska, USA	58°22' N	134°40' W	H11	Coyer	
CA-BC7	*Fucus* sp.	Bukleys Bay, British Columbia, CAN	49°32' N	124°51' W	H11	this study	KT306723
US-WA4	*Fucus* sp.	Sekui, Washington, USA	48°16' N	124°17' W		this study	KT306725
		**NF/GOM/GSL**					
AY659888	*F*. *distichus*	Appledore Is, Maine, USA	42°59' N	70°37' W	H9	Coyer	
AY659891	*F*. *distichus*	Appledore Is, Maine, USA	42°59' N	70°37' W	H5	Coyer	
US-ME8	*F*. *distichus*	Outer Gouldsboro Bay, Maine, USA	44°24' N	67°58' W	H5	this study	KT306708
CA-Nfld3	*F*. *distichus*	Cape Onion, Newfoundland, CAN	51°37' N	55°37' W	H5	this study	KT306707
US-ME9	*F*. *distichus*	Dry Is., Gouldsboro Bay, Maine, USA	44°25' N	67°58' W	H5	this study	KT306706
US-ME10	*Fucus* sp.	Goose Rocks Beach, Maine, USA	43°24' N	70°24'36'' W	H5	this study	KT306705
CA-Nfld4	*Fucus* sp.	Ferryland, Newfoundland, CAN	47°2' N	52°53' W	H3	this study	KT306694
CA-Nfld5	*Fucus* sp.	Witless Bay, Newfoundland, CAN	47°17' N	52°50' W	H2	this study	KT306702
CA-Nfld6	*Fucus sp*.	Heart's Desire, Newfoundland, CAN	47°49' N	53°26' W	H2	this study	KT306703
CA-Nfld7	*F*. *distichus*	Witless Bay, Newfoundland, CAN	47°17' N	52°50' W	H2	this study	KT306704
CA-Que1	*F*. *distichus*	Quapitogone Is., Quebec, CAN	50°12' N	60°6' W	H2	this study	KT306709
CA-Nfld8	*F*. *distichus*	Cape Onion,Newfoundland, CAN	51°37' N	55°37' W	H2	this study	KT306710
CA-Nfld9	*F*. *distichus*	Musgrave ledges, Newfoundland, CAN	49°27' N	53°58' W	H2	this study	KT306711
CA-Nfld10	*Fucus* sp.	Middle Cove, Newfoundland, CAN	47°39' N	52°42' W	H2	this study	KT306697
CA-Nfld11	*F*. *distichus*	Witless Bay, Newfoundland, CAN	47°17' N	52°50' W	H2	this study	KT306712
CA-Nfld12	*Fucus* sp.	Nameless Cove, Newfoundland, CAN	51°18' N	56°43' W	H4	this study	KT306687
CA-Nfld13	*F*. *distichus*	Heart's desire, Newfoundland, CAN	47°49' N	53° 26'	H2	this study	KT306686
AY941093	*F*. *gardneri*	Juneau, Alaska, USA	58°20' N	134°28' W		Coyer	
AY659886	*F*. *distichus*	Auke, Bay, Alaska, USA	58°22' N	134°40' W		Coyer	
AY659892	*F*. *distichus*	Arctic and Subarctic			H1	Coyer	
		**Fucus serratus**					
CA-NS1	*F*. *serratus*	Rose Point, Lunenburg Bay, Nova Scotia, CAN	44°17'36.5" N	64°13'52.4" W	nd	this study	KT306682
CA-NS2	*F*. *serratus*	St. Peters Bay, Pte du Loup, Nova Scotia, CAN	45°33'38" N	60°53'38.8" W	nd	this study	KT306683
CA-NS3	*F*. *serratus*	Shag Harbour, Lower Wood, Nova Scotia, CAN	43°30'35.9" N	65°44'14.2" W	nd	this study	KT306684
CA-NS4	*F*. *serratus*	Shag Harbour, Lower Wood, Nova Scotia, CAN	43°30'35.9" N	65°44'14.2" W	nd	this study	KT306680
CA-NS5	*F*. *serratus*	Cross Island, Nova Scotia, CAN	44°18'49" N	64°11'52.6" W	nd	this study	KT306681
AY659874	*F*. *serratus*	West European coast	NA	NA	nd	Coyer	
AY659876	*F*. *serratus*	West European coast	NA	NA	nd	Coyer	
AY659877	*F*. *serratus*	North Europe +Iceland	NA	NA	nd	Coyer	
AY659878	*F*. *serratus*	Brittany (Le Croisic), France	NA	NA	nd	Coyer	
AY659879	*F*. *serratus*	Brittany (Le Croisic), France	NA	NA	nd	Coyer	
AY659880	*F*. *serratus*	Oban, Scotland	NA	NA	nd	Coyer	
AY659881	*F*. *serratus*	Oban, Scotland	NA	NA	nd	Coyer	
AY659882	*F*. *serratus*	Oban, Scotland	NA	NA	nd	Coyer	
AY659883	*F*. *serratus*	North Atlantic	NA	NA	nd	Coyer	

After collection, samples were either prepared for molecular work or for mounting on herbarium sheets. When collection trips were longer than one week, subsamples of specimens were dehydrated using silica gel. During shorter expeditions, fresh samples were taken to the laboratory and immediately stored at 4°C. Over three days, these samples were individually rinsed three times in a 3% salt deionizing water solution to remove excess compounds and epiphytic organisms. A final rinse in an eliminating polysaccharide buffer wash consisting of 20% ethanol and 0.5 M potassium acetate (KAc) was conducted to eliminate excess polysaccharides [21]. Following this rinse, *Fucus* specimens were air-dried and a small portion was clipped from the main portion of each plant, ground in liquid nitrogen, and stored at -80°C for subsequent molecular analyses. The remaining algal material was then mounted on herbarium sheets for deposit in collections.

This research did not involve any work on human subjects, vertebrate animals, embryos or tissues. No permits were required because the collections were seaweeds and were not collected in Parks that had a marine sector. Collections were also not made on private land that required permission.

### DNA extraction and PCR amplification

DNA was extracted using a protocol developed by S.C. Hake (pers. comm.) based on previously published methods [21–22]. Approximately 1 cm^2^ pieces of dried tissue were placed in 2.0 mL screw capped microcentrifuge tubes with 0.1 mm diameter and 5.0 mm diameter glass and steel beads, respectively. The tissue was incubated in liquid nitrogen for 2 min before being pulverized in a Qiagen^®^ TissueLyser at 27 Hz for 90 seconds. To these tissue powder samples, 500 μL of extraction buffer (0.2 M Tris HCl, 0.25 M NaCl, 0.25 M EDTA, and 0.5% (w/v) SDS, at pH 7.5) preheated to 65°C, was added and vortexed. Due to the high viscosity of the resulting lysate solution, 50 μL of 20 mg/mL proteinase K was added. The solutions were incubated at 65°C for 30 min before being centrifuged to pellet the cell debris. The supernatant was transferred to a new tube with 173 μL 5.0 M NaCl and 400 μL of 100% isopropyl alcohol and gently mixed by inversion. Incubating the solutions at -20°C for 2 hours allowed the DNA to precipitate along with an equal mass of polysaccharides. The precipitate was pelleted and the pellet was washed with 70% ethanol before incubating at room temperature with an open lid inverted on a sterile cloth to allow all traces of ethanol to evaporate. 120 μL TE buffer was added to the pellet gently to not disrupt it, then incubated at room temperature overnight to allow the DNA to dissolve into the buffer and allow many of the polysaccharides to remain precipitated in the pellet.

Approximately 112 μL of the TE buffer was carefully removed so that the remaining pellet was not disturbed. To this, 56 μL 7.5 M ammonium acetate was added so that its final concentration was 2.5 M to remove any remaining polysaccharides. This solution was mixed with 340 μL of chilled ethanol to re-precipitate the DNA. The solution was incubated at -80°C for 1 hour before being centrifuged to pellet the DNA. The pellet was washed with 70% ethanol and then dried as described above. Again, 50 μL TE buffer was carefully added to the pellet and incubated undisturbed at 4°C overnight to allow the DNA to dissolve into the TE buffer, while the remaining polysaccharides were in the pellet. About 40 μl of extract was recovered and a 1:10 dilution of this was used as the template for PCR.

The amplification of the mitochondrial 23S ribosomal RNA gene-23S ribosomal RNA-tRNA- Val intergenic spacer region (23S mtDNA-IGS) was completed using forward primer 5‘-CGTTTGGCGAGAACCTTACC-3’ and reverse primer 5’- TACCACTGAGTTATTGCTCCC -3’ [11]. The final reaction solution volumes were 25 μl with 2.5 μl of Bioline Ammonium Buffer (10x), 2.0 μL of dNTP mix (10mM), 1.25 μL of magnesium chloride (50 mM), 1.0 μL of each primer (10 uM), 0.5 μL of Bovine Serum Albumin (10 mg/mL), 14.05 μL of nuclease-free water, 0.2 μL of BIOLASE^™^ Taq Polymerase (5U/uL), and 2.5 μL of DNA template (~20 ng/μL). PCR conditions adopted were 95°C for 3 min, followed by 35 cycles of 94°C for 40 sec, 51°C for 40 sec and 72°C for 1 min 40 sec, with 10 min of final elongation on 72°C followed by an 11°C soak. The amplification of the nuclear rDNA internal transcribed spacer region (ITS-1) fragment was conducted as described by Serrão et al. [23]. Due to intraindividual polymorphisms in the nrDNA-ITS, we cloned several nuclear rDNA-ITS PCR products (TOPO^®^ TA Cloning^®^ kit, Life Technologies^™^). Plasmid DNA from each recombinant colony was extracted afterwards. PCRs were performed on an Eppendorf Mastercycler^®^ Gradient 5331 (Eppendorf, Canada) and a BioRad C1000 Touch^™^ thermal cycler (BioRad, USA).

PCR products (23S mtDNA-IGS) were separated by electrophoresis using an agarose gel 1.5% and treated with 2 μL of 1:3 diluted ExoSapIT^®^ enzyme mixture (Affymetrix-USB) before being used as a template for cycle sequencing. For each sample, 8μl of a cycle sequencing reaction mixture (including 0.8 μL BigDye^®^ [Terminator 3.1, Applied Biosystems^®^]) 1.0 μL 1 μM primer, 2.0 μL 5x buffer, 3.7 μL water, and 0.5 μL dimethyl sulfoxide) was combined with 4μL of treated PCR product. The reactions were conducted in a BioRad C1000 thermal cycler in which there were 30 cycles of 95°C for 30 sec, 55°C for 30 sec and 60°C for 4 min. The cycle sequencing products were purified using Sephadex G-50 (GE HealthCare) columns, dehydrated, and re-suspended in HiDi formamide (Life Technologies^™^). The sequences were obtained from an ABI 3730xl automated capillary sequencer with a 50 cm 96 channel array with Pop-7 polymer. The chromatograms were manually edited using Sequencher^®^ v 2.1 (Gene Codes Corp., Ann Arbor, MI, USA).

### Phylogenetic and evolutionary analysis

Sequences were aligned using MUSCLE [24], then manually refined using Geneious software [25]. For construction of the phylogenetic trees, substitution models for nucleotide evolution for both the mtDNA-IGS and the nrDNA-ITS were determined using a model testing software implemented in MEGA6 software [26]. Under the Akaike Information Criterion (AIC) (jModelTest–[27]), the TN92 model was utilized for the mtDNA-IGS and TN93 + gamma for the nrDNA-ITS.

The final mtDNA-IGS dataset was composed of 74 sequences from *Fucus distichus*, *F*. *serratus* L, and *F*. *vesiculosus* (NC_007683). Neighbor Joining (NJ), Maximum Likelihood (ML), and Maximum Parsimony (MP) analyses were conducted using MEGA6 software [26].

For the neighbor joining method, the optimal tree with the sum of branch length = 0.18843535 is shown in supplementary material. For the maximum likelihood method, the tree with the highest log likelihood (-1375.7931) was chosen. The percentage of trees in which the associated taxa clustered together is shown next to the branches. Initial tree(s) for the heuristic search were obtained by applying the Neighbor-Joining method to a matrix of pairwise distances estimated using the Maximum Composite Likelihood (MCL) approach. The tree is drawn to scale, with branch lengths measured in the number of substitutions per site. For the maximum parsimony method, the first tree out of 10 most parsimonious trees (length = 97) was selected. The consistency index was (0.833333), the retention index was (0.975543), and the composite index was 0.884086 (0.812953) for all sites and parsimony-informative sites (in parentheses). The percentage of replicate trees in which the associated taxa clustered together in the bootstrap test (1000 replicates) is shown next to the branches in the supplementary material. The MP tree was obtained using the Subtree-Pruning-Regrafting (SPR) algorithm with search level 1 in which the initial trees were obtained by the random addition of sequences (10 replicates).

In the rDNA-ITS, 474 sequences were used (S1 Fig, S1 Table). Neighbor Joining (NJ) and Maximum Likelihood (ML) were conducted using MEGA6 software [26] and Bayesian [28] (data not shown) and RaxML through the CIPRES Gateway [29], respectively. Statistical analyses of tree topologies were conducting using 1000 pseudoreplicates. The tree is drawn to scale, with branch lengths measured in the number of substitutions per site.

A statistical parsimony network with 95% connection limits was constructed using the mtDNA-IGS sequences with the program TCS v. 1.21 [30]. Sequences were submitted to GenBank and accession numbers are included in Table 1.

## Results

### Species Identification

**Fig 2:**
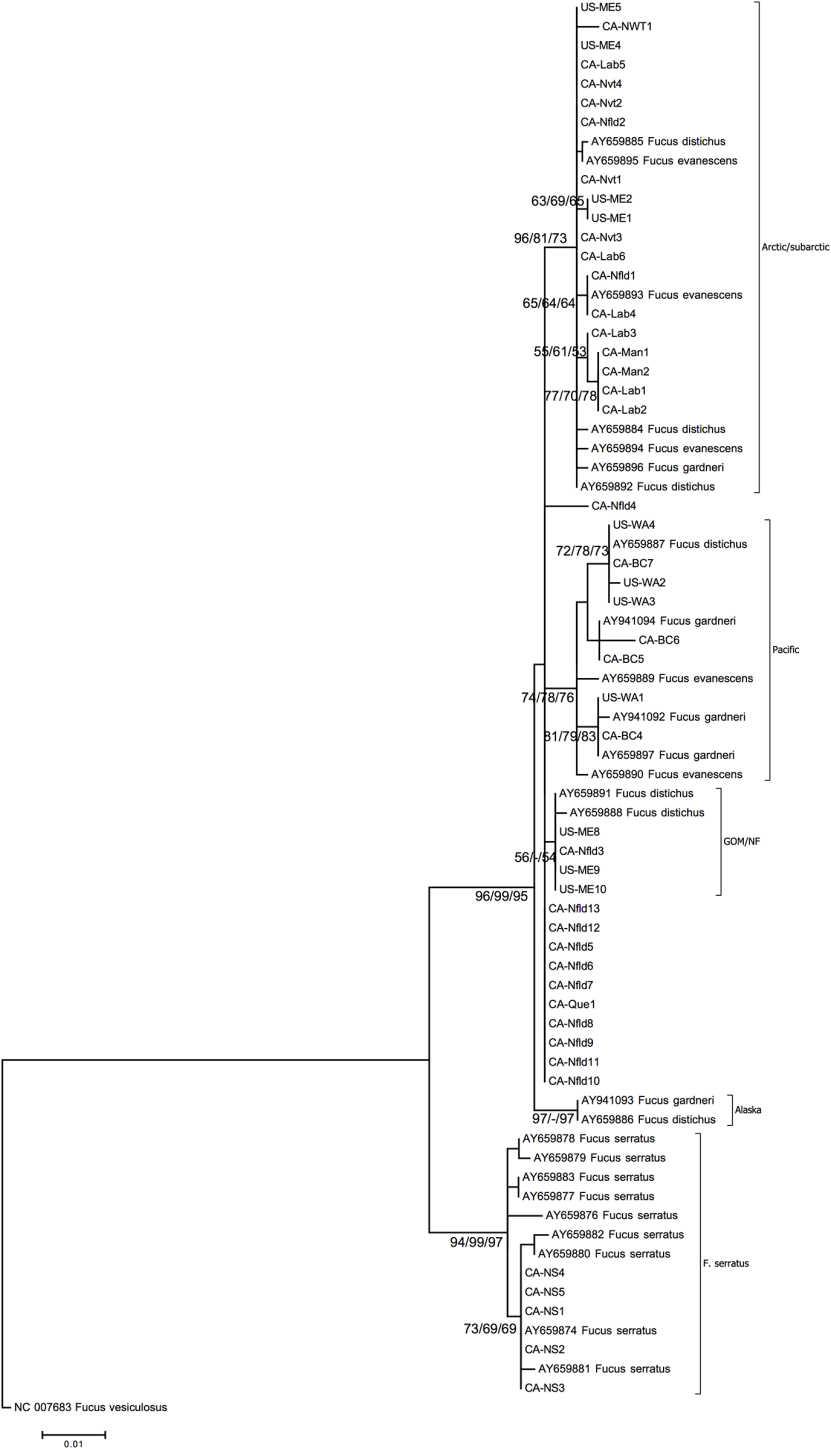
Maximum Likelihood Tree based on mtDNA-IGS for *Fucus distichus*, *F*. *evanescens*, *F*. *garderni*, and *F serratus* specimens from this study and from GenBank (Table 1). This tree includes neighbor-joining, maximum parsimony, and maximum likelihood bootstrap replicates (1000 replicates); over 50% are indicated at the nodes. The sequence of *F*. *vesiculosus* (NC_007683) is used as an outgroup.

Specimens were classified using morphological characteristics according to Sears [20]. The identification was also supported by the mtDNA-IGS (Fig 2) and the nrDNA-ITS (S1 Fig). Fifty-two specimens of *F*. *distichus* were collected by the authors and utilized in this study. Only a single specimen was shown not to be *F*. *distichus*, but rather *F*. *vesiculosus*, by molecular methods; however, the specimen had been labelled *questionable*.

### mtDNA intergenic spacer (mtDNA-IGS)

The mtDNA intergenic spacer was sufficient for species-level separation between *Fucus distichus* and *F*. *serratus*; however, *F*. *evanescens* and *F*. *gardneri* were not separated from *F*. *distichus* with this marker (*F*. *vesiculosus* was used as the outgroup). This marker was also sufficient for demonstrating the biogeographic patterns in both *F*. *distichus* and *F*. *serratus* (Fig 2). Altogether, 59 sequences obtained from specimens of *F*. *distichus* and 14 sequences of *F*. *serratus* originating from the North Pacific to North Atlantic were analyzed using phylogeny and a statistical parsimony network. These sequences complement earlier studies undertaken [7,11].

The maximum likelihood analysis resulted in one maximum likelihood tree (log likelihood = -1375.7931) (Fig 2) that revealed four distinct clades: Arctic/Subarctic, Gulf of Maine/Newfoundland, North Pacific, and two sequences from Alaska with some unresolved branches including sequences originating from Newfoundland and Québec. Thus the Arctic/Subarctic, Gulf of Maine/Newfoundland, North Pacific and Alaska seaweed clades were supported by bootstrap values higher than 50%. The Arctic/Subarctic and North Pacific clades included some smaller subclades (i.e. diversified genotypes). The unresolved branches had uncertain positions in the tree, without any significant bootstrap values, from different areas in Newfoundland and Québec.

**Fig 3:**
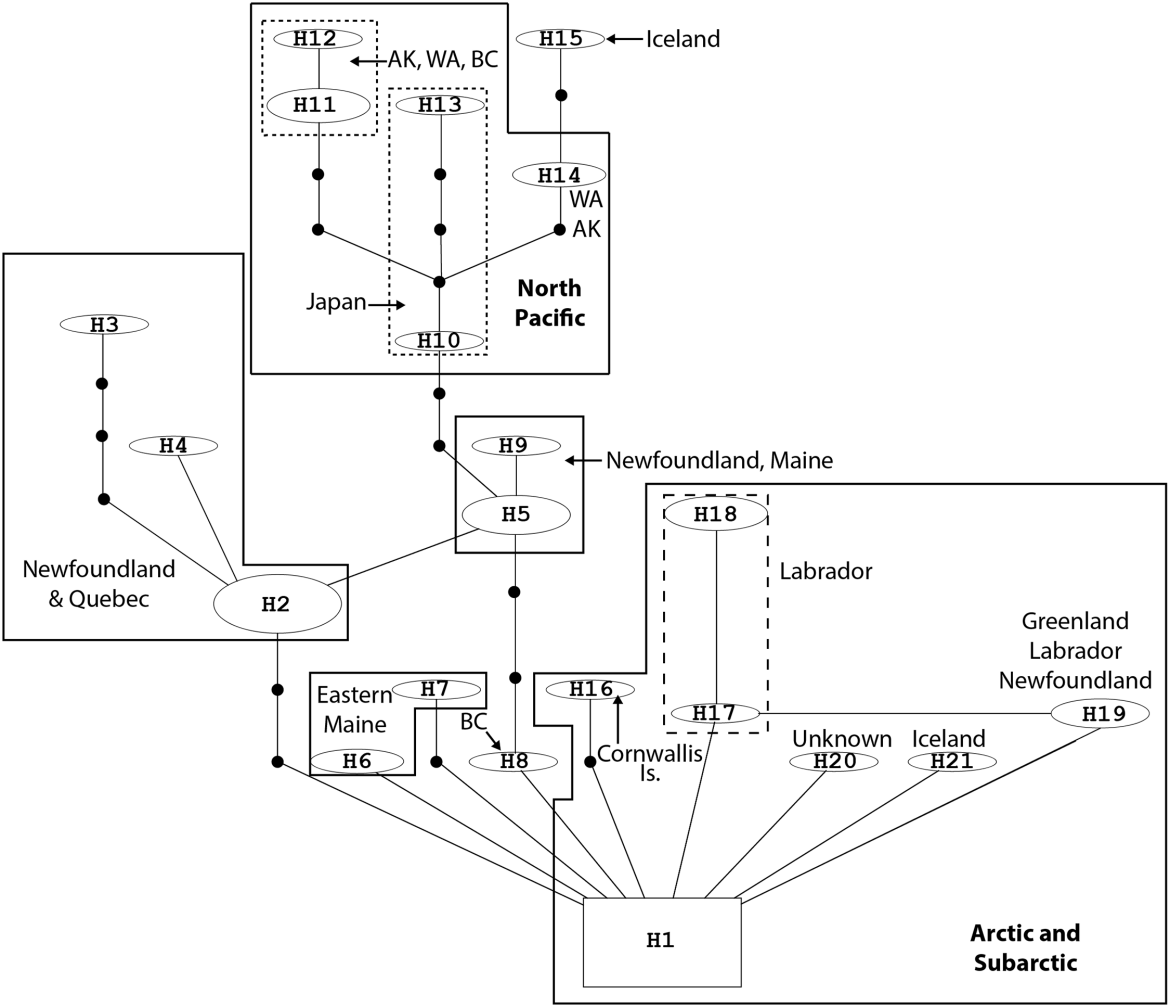
Statistical parsimony network showing relationships among 21 mtDNA-IGS haplotypes of the genus *Fucus* (Table 1) in North America. The square box represents the haplotype that the program TCS hypothesized as the ancestral haplotype. Solid circles indicate mutational steps, solid line also indicates a mutational step.

The statistical parsimony network analysis examined 58 mtDNA-IGS sequences from *Fucus distichus*, *F*. *evanescens* and *F*. *gardneri*. This resulted in one large network containing 21 mtDNA-IGS haplotypes from 53 mtDNA-IGS sequences (Table 1, Fig 3, S1 Fig). Two sequences (CA-BC4 and US-WA4) were not connected to the larger network and had large indels in the middle of the mtDNA-IGS region. The remaining 3 unconnected sequences formed a small network of two haplotypes (CA-BC6, CA-BC5 and AY941094; not shown) and are from the Pacific and group with other Pacific collections in the mtDNA-IGS tree. In examination of the alignment, these sequences three share two indels with the sequences of *F*. *serratus* from this study and those of Coyer [7,11]. The first is an 8-nucleotide indel near the 5’ end and the second is a 48 nt indel that begins at the same point of the 5’ end of a 111 nt indel that is observed in some *F*. *serratus* sequences. These nine *F*. *serratus* sequences also formed a small network of four haplotypes, all of which contained both the 8 nt and the 111 nt indel. There was also a smaller network of *F*. *serratus* containing three haplotypes and four sequences (AY659883, AY659877, AY659878, AY659879; not shown). The sequences AY659878 and AY659879 did not contain either of the indels noted previously but the other two sequences contained the first 8 nt indel.

The larger network of 21 haplotypes from *Fucus distichus*, *F*. *evanescens* and *F*. *gardneri* corresponds to the Arctic/Subarctic, GOM/NF and North Pacific clades seen in Fig 2, and also includes sequences that are unresolved as being part of these three clades, yet are still grouping within a larger clade that includes these three groups. The H1 haplotype contains 11 mtDNA-IGS sequences, which appear to be Arctic/Subarctic in origin (Fig 3, S1 Fig) and was determined by the program TCS as the likely ancestral haplotype. Haplotype, H2 contains eight mtDNA-IGS sequences from the Avalon and Northern Peninsula of Newfoundland and one from Québec and has two additional haplotypes that are separated by a number of mutational steps (Table 1, Fig 3, S1 Fig). For instance, H3 is separated from H2 by 4 mutational steps and H4 is separated from H2 by 1 mutational step (Fig 3). Haplotypes H3 and H4 include sequences from Newfoundland that are within the geographic range of H2. There are a number of haplotypes that are derived from the Arctic/Subarctic haplotype (H1) and include sequences from the North Atlantic: Maine (H5-H9), Newfoundland (H2, H5, H19), Labrador (H17-H19), Cornwallis Island (H16), Greenland (H19), and Iceland (H21) (Fig 3). Haplotype H8 also appears to be derived from the Arctic/Subarctic haplotype and contains one mtDNA-IGS sequence from southern British Columbia; this is then connected to haplotype H5 via three mutational steps. The latter haplotype contains sequences that are North Atlantic in origin (Maine, Newfoundland) and form a loop with haplotype H2, which represents uncertainty. Populations that are Pacific in origin, with the exception of H8, are derived from H5 and are separated by a minimum of two mutational steps and include collections from Japan (H10, H13), Washington (H11, H12, H14), Alaska (H11, H14), and British Columbia (H11, H14). The one exception in this group is H15, which is a collection of *F*. *distichus* from Iceland.

### Internal Transcribed spacer region of nuclear ribosomal DNA (nrDNA-ITS)

The nrDNA-ITS was sufficient for species-level separation among *Fucus distichus*, *F*. *serratus*, and *F*. *vesiculosus* supporting our mtDNA-IGS results, which separated these species, and showed that *F*. *evanescens* and *F*. *gardneri* are not distinct species separated from *Fucus distichus*. However, this marker did not resolve biogeographic nor population-level patterns in *F*. *distichus*, as observed with the mtDNA-IGS. Furthermore, we also observed several copies of the nrDNA-ITS (through cloning), which have also been noted by other authors [23]. Interestingly, this marker did not support the separation of *F*. *ceranoides*, *F*. *spiralis*, *F*. *vesiculosus*, and *F*. *virsoides*. We present the data in S2 Fig, but will not discuss them in detail in this study, since our focus is on the more useful information from the mtDNA on both biogeographic and population-level patterns and the haplotype analyses.

## Discussion

Although *F*. *distichus* is widely distributed along rocky shores of the colder North Atlantic, its specific biogeography in the northwestern Atlantic and Arctic/Subarctic zones has not been sufficiently studied due to the challenges of sampling in these regions. Based on mtDNA-IGS data, Coyer et al. [7] proposed that *F*. *distichus* likely had two colonization events from the North Pacific to the Atlantic since the opening of the Bering Strait. The first from the NW Pacific and the other was from the NE Pacific (Gulf of Alaska). However, Cánovas et al. [31] predicted that while Fucaceae originated in the Pacific, *F*. *distichus* had a putative Atlantic/Arctic ancestor (on the same side of the Bering Strait), with a later invasion to the Pacific. Based on our analyses, the situation is likely far more complex; ancestral *F*. *distichus* is based in the Arctic and Subarctic, and the North Pacific ancestor is unknown.

Our analyses using the mtDNA-IGS showed that there were four distinct clades of *F*. *distichus* between the NE Pacific and NW Atlantic. *Fucus distichus* consisting of the four subclades and some unresolved tips (Fig 2) is monophyletic. The three larger subclades in these four clades are polyphyletic with GenBank sequences from *F*. *gardneri* and *F*. *evanescens*. Studies by Coyer et al. [11] and Cánovas et al. [31] with the same species support this polyphyletic relationship. Traditional bifurcating tree-building algorithms often do not resolve closely related evolutionary histories at the population level [30–32]. Statistical parsimony networks do not assume a bifurcating pattern of ancestor and descendant relationships but groups very closely related haplotypes and has been applied frequently to address biogeographic questions among closely related organisms including pygmy whitefish [33], marine gastropods [34] and many other organisms. The network from this study (Fig 3) showed that mtDNA-IGS sequences from *F*. *distichus*, *F*. *evanescens* and *F*. *gardneri* all form a related network. The only exception were two sequences (CA-BC4 and US-WA4) that had large indels or missing data yet still appeared to cluster with the other mtDNA-IGS sequences of these species in the maximum likelihood tree (Fig 2). The structure of the maximum likelihood tree (Fig 2) also does not support the separation of the three *Fucus* species (*F*. *distichus*, *F*. *evanescens*, and *F*. *gardneri*). The short evolutionary distance, from the node to the most distal sample, indicates very little evolutionary change among samples within the clades. The combination of these three species into a single *F*. *distichus* has been well supported in the literature for over 30 years [11,23,31,35–38].

It has long been recognized that the last interglacial (the Sangamonian or Eemian Interglacial) was considerably warmer, and with a sea level roughly 8 m higher than at present [39]. More recent evidence from Antarctic ice cores suggest that several of the earlier Pleistocene interglacials had equivalently higher temperatures [40–41], although early Pleistocene temperature variations were probably more moderate and with a different time frequency. It is possible that the link of the entire North Pacific set of haplotypes with some Newfoundland samples and the high Arctic haplotype represents a residual of reproductively continuous and more compact *Fucus distichus* populations from the warmer last interglacial, or perhaps also the previous one or two interglacials. The Thermogeographic Model [5] demonstrated that a true Arctic flora of seaweeds is likely to be very limited, as the area available to Arctic species becomes minimal during glacial epochs. It is the Subarctic flora that highly dominates, occupying much of the rocky shore in the Canadian North that we typically refer to as Arctic. In this paper, Subarctic and Arctic are not distinguished as separate biogeographic regions. Additional sampling from both Newfoundland, with its exceptional diversity of *F*. *distichus* genotypes (unresolved tips), and the high Arctic will likely provide more information on this subject.

The taxonomic separation between *F*. *distichus*, *F*. *evanescens*, and *F*. *gardneri* was largely based on habitat [8,42]. *Fucus gardneri*, described as an endemic species of the North West Pacific [17,23], is not distinguishable as a species using neither mtDNA nor nrDNA with phylogenetic analyses. This species was erected as a *nomen novum* for *F*. *furcatus*, since it was a *nomen illegitimum* by Silva [42]. Furthermore, the overlapping habitat between *F*. *distichus* and *F*. *gardneri* has been reported in the North Pacific and Pacific coasts of Alaska [43–46]. In addition, reproductive structures and methods are nearly identical across all three cited species providing further evidence for the lack of support for the separation of these three species. All three species, *F*. *distichus*, *F*. *evanescens*, and *F*. *gardneri* are monoecious with both male and female gametes present in conceptacles of a single plant and with recorded hybridizations across all three species in both laboratory and natural settings [16,23,36]. In addition, the region of growth within the intertidal zone has substantial overlap across all three species; with this large overlap in the near shore habitat, hybridization between species is probable.

The continued acceptance of *F*. *distichus*, *F*. *evanescens*, and *F*. *gardneri* based on available literature is not supported by any of the following species concepts. The data concerning these species (Fig 2) is not supported by the phylogenetic species concept due to the lack of support from resampling methods and lack of monophyletic clades representing each individual species. The biological species concept is not supported because the data indicate no distinct reproductive barriers, either due to incompatible sexual characteristics or physical barriers among the three species. The ecological species does not provide sufficient cause to keep all three species separated, as all three species are located on temperate rocky intertidal shores. Finally, the morphological species concept is not valid in distinguishing these species based on measured characters, and therefore is not viable as a concept among *Fucus distichus* varieties (due to plasticity among the varieties).

The variations within the clades (Fig 2) do not support separation of species, however, there are clear biogeographic trends forming localized populations. For example, there are three distinct groups of samples with a geographic relationship (and 2 tips in an Alaskan clade). The first group is the North Pacific clade. This clade contains the samples from several locations in Washington (USA) and British Columbia (Canada); all collected from the northeastern Pacific shores. The Arctic and Subarctic Clade is comprised of samples collected from shores of the Manitoba, Northwest Territories, Nunavut, and northern Newfoundland and Labrador (Canada). The final region sharing a cluster is the region of the Gulf of Maine and southern Newfoundland. Interestingly, the North Pacific clade shares a more recent common ancestor with this latter clade than with the Arctic/Subarctic. This is similar to the mtDNA-IGS network, which clearly showed similar biogeographic groupings in the North Atlantic, Pacific and Arctic. It appears that there is a distinct Arctic haplotype that may be the source of haplotypes in the North Pacific and North Atlantic (Fig 3) and haplotypes from Labrador, Greenland, and Iceland are closely associated with this group.

During the last glacial period (12 000 to 18 000 BP) Cornwallis and Beechey Islands were ice-free and were potential refugia for this species [15]. It is likely that other closely related Arctic populations were forced southward into both the North Atlantic and Pacific providing gene flow to subpopulations in those regions. In addition, Newfoundland appears to have divergent haplotypes (five different haplotypes are present) that also may be ancestral in origin; however, this region needs to be sampled more extensively. These divergent haplotypes may also be explained by ice-free refugia seen in Newfoundland in the same time periods noted for Beechey and Cornwallis islands [15]. For example, 18 000 years BP, small portions of Newfoundland (now including the exposed Grand Bank area) were unglaciated and isolated [15]. Haplotype H3, which is separated by three mutational steps from haplotype H2, is a population from Ferryland on the Avalon Peninsula (Newfoundland) and is in a region that would have remained unglaciated but separated from the H2 haplotype. There are haplotypes in Maine that are between 1 and 4 mutational steps separated from the Arctic core population and these may represent relics from recent glaciations that are maintained due to the cold conditions in eastern Maine and southwestern Nova Scotia, deriving from strong tidal mixing. The haplotype in Iceland that is associated with the Pacific clade is likely due to vector-assisted transport via human activity.

The unresolved branches in the mtDNA phylogeny are unusual in showing widely separate populations around Newfoundland. While the collection of additional samples is needed to clarify this situation, it is not entirely unexpected. As described by Adey and Hayek [14], the early Holocene likely provided continuous population connection and gene flow across the lower Canadian Arctic Archipelago. We assume tentatively that this population is a relic from that period, 6000–8000 years BP. With clear biogeographic trends present among *F*. *distichus* samples, the strict application of the maximum likelihood hypothetical evolutionary metric is well supported with clades belonging to entirely specific regions. Relationships among Pacific, Arctic, and Atlantic Oceans, are biogeographically supported in the maximum likelihood phylogenetic tree with the separation of the different clades. Finally, seasonal reproductive variation among *F*. *distichus* (reported among *F*. *evanescens*, *F*. *gardneri*, and *F*. *distichus*) [11,23] can contribute to genetic isolation among populations, which can lead to distinct biogeographic groups. The presence (Figs 2, 3) of these clearly defined biogeographic patterns further supports the recognition of a single *F*. *distichus* species influenced by populations in different biogeographic regions.

Due to the conclusions noted above, and conclusions by other authors [7,11,31], we propose to synonymize *F*. *evanescens* and *F*. *gardneri* with *F*. *distichus*. However, several distinct regional variants are clearly present (Fig 2). This strongly suggests periods of geographic separation during glacial periods leading to the development of regional variation. Since the regional variants are reconnected during interglacials and *Fucus distichus* is monoecious in its sexual reproduction, re-mixing has kept further divergence from occurring as it has done in other clades of the Fucaceae. On the other hand, *F*. *distichus* is the only species of the Fucaceae to develop Arctic and Subarctic capabilities; in its low intertidal and uppermost sublittoral habitat, this likely relates primarily the ability of this species to withstand long periods each year of sea ice cover. Thus, unlike the other species within the Fucaceae that subsequently evolved to fit warmer climes, populations of *F*. *distichus* in the North Atlantic and North Pacific were frequently linked for longer times during interglacial periods and form a continuum of populations throughout the Arctic/Subarctic.

### New Synonym

#### *Fucus distichus* Linnaeus

Linnaeus, C. (1753). Species plantarum, exhibentes plantas rite cognitas, ad genera relatas, cum differentiis specificis, nominibus trivialibus, synonymis selectis, locis natalibus, secundum systema sexuale digestas. Vol. 2 pp. [i], 561–1200, [1–30, index], [i, err.]. Holmiae [Stockholm]: Impensis Laurentii Salvii.

Locations: Canadian Arctic, Norway, North America (Maine, Massachusetts, and New Hampshire), Alaska.

### Homotypic Synonyms

#### *Fucus evanescens* C. Agardh

C. Agardh (1820) in Agardh, C.A. (1820 '1821'). Species algarum rite cognitae, cum synonymis, differentiis specificis et descriptionibus succinctis. Volumen primum. Pars prima. pp. [i-iv], [1]-168. Lundae [Lund]: ex officina Berlingiana.

Location: Canadian Arctic, North American East Coast, Iceland, Norway.

#### *Fucus gardneri*

PC Silva (1953) in Silva, P.C. (1953). The identity of certain Fuci of Esper. Wasmann Journal of Biology 11: 221–232.

Location: British Columbia (Canada); Alaska, Oregon, Washington (USA)

## Conclusions

Several authors have noted that the ancestor of *Fucus distichus* evolved in the North Pacific in the late Tertiary and that other shallow water Arctic and Subarctic biota are largely based in the Tertiary North Pacific [see eg. 5, 6]. However, our haplotype network, with an extensive sampling in these regions, clearly shows the ancestor of the *Fucus distichus* complex to be centered in the low Arctic and Subarctic. It seems likely that during each interglacial cycle, the Canadian Arctic Archipelago, and its Subarctic fringes, became the central “mixing bowl” and the prime genetic repository for this low intertidal, sea ice-adapted species. With every cycle of glaciation, this core population has moved to the northernmost regions of the northwestern North Atlantic and North Pacific and the occupied geographical area became reduced [5]. Thus, the current subspecies populations, each slowly developing distinctive localized characters and physiological capabilities, interbreed and become genetically diluted with genes from the ancestral Arctic/Subarctic population during each glacial cycle. The very distinctive character of the ancestral subspecies, based on a physiological adaptation from three to nine months of sea-ice cover and disturbance, is not required in the peripheral subspecies, but clearly provides an advantage to hybrids that maintain the reproductive and population link. The features of the ancestral species of *F*. *distichus* were selected to allow this entity to become a dominant player in the most extreme part of the Arctic, the lower intertidal. This was maintained throughout the enormous area of expansion available during each Interglacial cycle, whereas the peripheral subspecies occupy areas that are smaller and more geographically fragmented at all times.

## Supporting Information

S1 Fig Map of North America focused on higher latitudes showing the distribution of haplotypes observed (Fig 3) in each oceanic basin with approximate locations, with the exception of Western Pacific locations (Japan). Exact locations for samples are noted in Table 1.
(TIF)

S2 Fig Neighbor-joining Tree based on the nrDNA-ITS for *Fucus ceranoides*, *F*. *distichus*, *F*. *evanescens*, *F*. *garderni*, *F serratus*, *F*. *spiralis*, *F*. *vesiculosus*, and *F*. *virsoides* specimens from this study and from GenBank (S1 Table). This tree includes several clones (cln_letter) form each plant at each locality we sampled. The tree includes neighbor-joining bootstrap replicates (1000 replicates), with those over 50% indicated on the nodes.
(PDF)

S1 Table List of taxa, collector, accession number, and name used in the nrDNA-ITS tree (S2 Fig). Since each of our specimens has several clones, the list of accession number that includes all the clones is provided for each sample locality. More information on longitude, latitude, and sampling location can be verified in Table 1 or in the manuscripts Kucera & Saunders (2008) Botany 86:1065–1079 and/or Serrão et al. (1999) J.Phycol. 35: 382–394.
(XLSX)
