# Supplementary figures and images for: Evolution of the Northern Rockweed, Fucus distichus, in a Regime of Glacial Cycling: Implications for Benthic Algal Phylogenetics
Source: PLoS One. 2015 Dec 2;10(12):e0143795. doi: 10.1371/journal.pone.0143795 (PMC4668022; doi:10.1371/journal.pone.0143795)

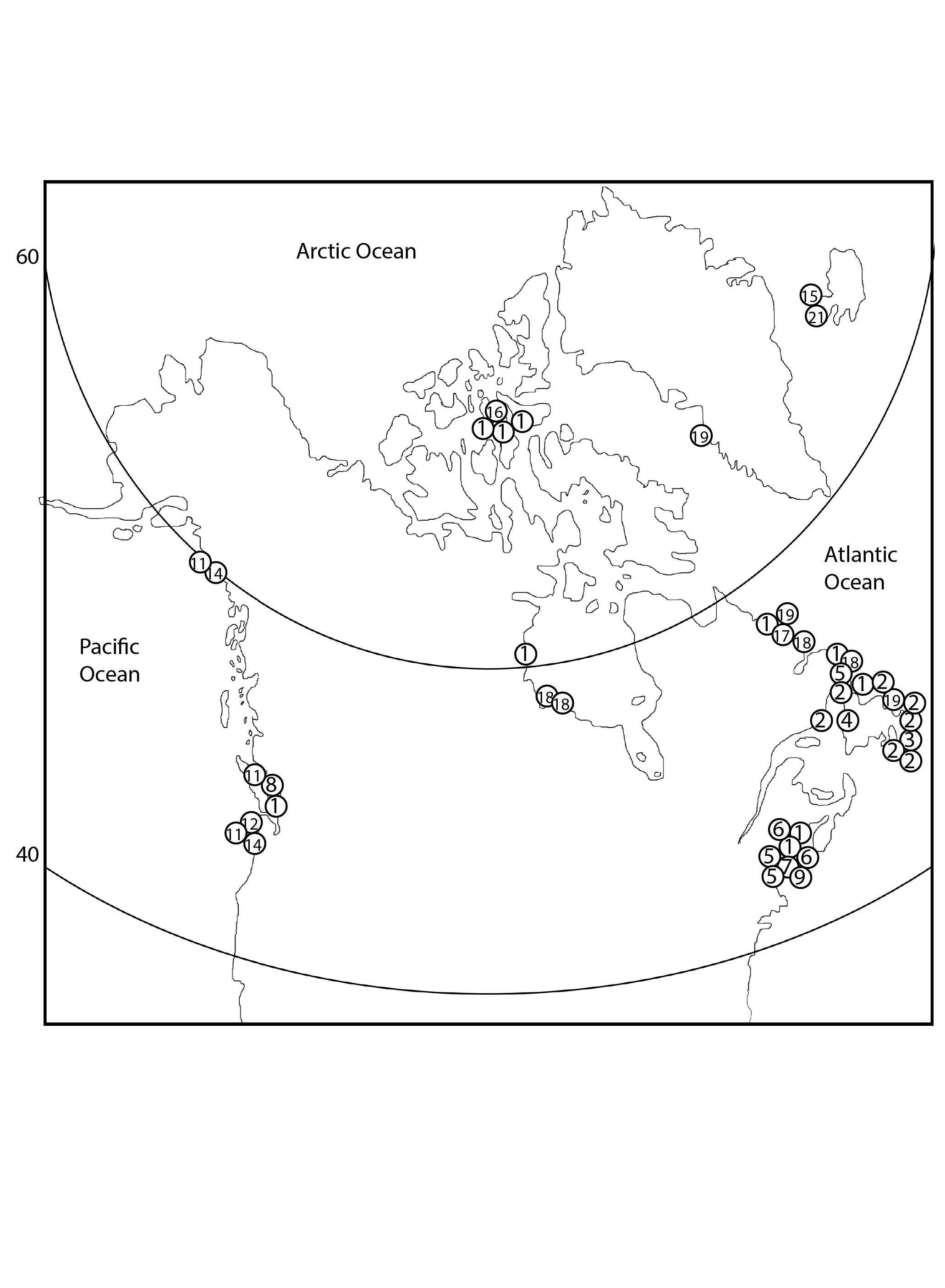

Supplement: S1 Fig — Exact locations for samples are noted in Table 1. (TIF) [file pone.0143795.s001.tif]

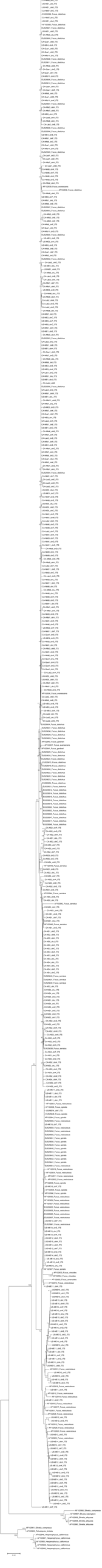

Supplement: S2 Fig — This tree includes several clones (cln_letter) form each plant at each locality we sampled. The tree includes neighbor-joining bootstrap replicates (1000 replicates), with those over 50% indicated on the nodes. (PDF) [file pone.0143795.s002.pdf]
